# The Association Between Ascending Aortic and Left Ventricular Dimensions in Patients After Aortic Valve Replacement

**DOI:** 10.21470/1678-9741-2023-0221

**Published:** 2024-02-21

**Authors:** Ingrid Schusterova, Panagiotis Artemiou, Ivo Gasparovic, Tibor Poruban, Marianna Barbierik Vachalcova, Karolina Angela Sieradzka, Silvia Gurbalova, Pavol Zenuch

**Affiliations:** 1 First Cardiological Clinic, East Slovak Institute of Cardiovascular Diseases, Pavol Jozef Safarik University, Kosice, Slovakia; 2 Clinic of Cardiac Surgery, National Institute of Cardiovascular Diseases, Faculty of Medicine, Comenius University, Bratislava, Slovakia

**Keywords:** Aortic Size, Aortic Valve Replacement, Ascending Aorta, End-Diastolic Diameter, Left Ventricle

## Abstract

**Introduction:**

Aortic valve replacement (AVR) is often recommended for patients with severe aortic stenosis or chronic aortic regurgitation. These conditions result in remodeling of the left ventricle, including increased interstitial fibrosis that may persist even after AVR. These structural changes impact left ventricular (LV) mechanics, causing compromised LV diameter to occur earlier than reduced LV ejection fraction (LVEF). The aim of this study was to examine the effect of left ventricular end-diastolic diameter (LVEDD) and its role in aortic expansion one year after AVR.

**Methods:**

Sixty-three patients who underwent AVR were evaluated. All patients underwent standard transthoracic echocardiography, which included measurements of the ascending aorta, aortic root, LVEF, and LVEDD before the surgery and one year postoperatively. Correlations between these variables were calculated.

**Results:**

All patients underwent AVR with either a mechanical or biological prosthetic aortic valve. Following AVR, there was a significant decrease in the dimensions of the ascending aorta and aortic root (both P=0.001). However, no significant changes were observed in LVEDD and LVEF. Correlations were found between the preoperative ascending aortic size and the preoperative and one-year postoperative LVEDD (r=0.419, P=0.001 and r=0.320, P=0.314, respectively). Additionally, there was a correlation between the postoperative ascending aortic size and the preoperative and one-year postoperative LVEDD (r=0.320, P=0.003 and r=0.136, P=0.335, respectively).

**Conclusion:**

The study findings demonstrate a significant correlation between the size of the aortic root and ascending aorta, before and after AVR. Additionally, a notable correlation was observed between postoperative LVEDD and the size of the aortic root.

**Table:** 

Abbreviations, Acronyms & Symbols				
AoAa	= Ascending aortic dimension before operation		IVS	= Interventricular septum
AoAb	= Ascending aortic dimension one year after operation		LV	= Left ventricular
AoBa	= Aortic bulb dimension before operation		LVEDD	= Left ventricular end-diastolic diameter
AoBb	= Aortic bulb dimension one year after operation		LVEDDa	= Left ventricular end-diastolic diameter before operation
AR	= Aortic regurgitation		LVEDDb	= Left ventricular end-diastolic diameter one year after operation
AS	= Aortic stenosis		LVEF	= Left ventricular ejection fraction
AVR	= Aortic valve replacement		LVEFa	= Left ventricular ejection fraction before operation
BAV	= Bicuspid aortic valve		LVEFb	= Left ventricular ejection fraction one year after operation
CABG	= Coronary artery bypass grafting		LVESD	= Left ventricular end-systolic diameter
EF	= Ejection fraction		TAV	= Tricuspid aortic valve

## INTRODUCTION

When patients experience symptoms of left ventricular (LV) dysfunction due to severe aortic stenosis (AS) or severe chronic aortic regurgitation (AR), it is recommended to undergo aortic valve replacement (AVR)^[1,2]^. AS and AR are two valvular heart diseases with distinct pathophysiologies and differ in the progression of LV remodeling and symptom development. AS puts pressure overload on the left ventricle, while AR causes both pressure and volume overload. These abnormal hemodynamic conditions lead to different responses in LV remodeling: AS results in concentric hypertrophy through increased muscle fiber diameter and the addition of new myofibrils in parallel, whereas AR leads to eccentric remodeling and LV dilation through the growth of cardiomyocytes and the addition of new sarcomeres in series^[3,4]^. In both cases, interstitial fibrosis tends to increase, which may persist even after relief from volume and/or pressure overload following AVR. These structural changes affect LV mechanics, and although LV ejection fraction (LVEF) may remain preserved for a considerable period, LV diameter may be compromised at earlier stages. As a result, patients with severe AS or AR may tolerate the volume overload state for many years and remain asymptomatic even after the development of LV dilatation and dysfunction^[5]^.

AVR is an effective treatment for patients with severe AS or AR. According to the current guidelines from the American Heart Association/American College of Cardiology and the European Society of Cardiology/European Society for Cardio-Thoracic Surgery, intervention is recommended for symptomatic patients with severe high-gradient AS or severe low-flow, low-gradient AS with reduced ejection fraction (EF) (< 50%) and evidence of flow (contractile) reserve. Additionally, asymptomatic patients with severe AS and systolic LV dysfunction (LVEF < 50%) without another cause or demonstrable symptoms on exercise testing should undergo AVR^[1,2]^. For patients with severe chronic AR, AVR is recommended if they have symptoms and/or LV dysfunction (EF < 50%), LV end-diastolic diameter (LVEDD) > 65 or 70 mm, and/or LV end-systolic diameter > 50 mm^[1,2]^. Several studies have investigated the ability of AVR to correct hemodynamic disturbances in AR patients with significantly dilated left ventricle and achieve postoperative LV reverse remodeling^[6,8]^.

Reverse remodeling is a process observed in AS patients after valve replacement, characterized by initial hypertrophy followed by regression of ventricular mass and improved ventricular function. This positive change can be assessed using echocardiograms or magnetic resonance imaging^[9]^. The most significant reduction typically occurs within the first six months but continues to improve for up to two years after surgery. This remodeling is characterized by a decrease in the LV mass/volume ratio, reduction in cavitary volumes, and improved diastolic filling and overall heart function^[10]^. The factors influencing LV reverse remodeling and outcomes after AVR for severe LV dilatation and systolic dysfunction have not been extensively researched.

In this study, we conducted the first evaluation to determine the impact of LVEDD on aortic expansion following AVR one year after the procedure.

### Objective

Several studies have investigated the outcomes of the ascending aorta and aortic root following AVR, exploring factors such as bicuspid aortic valve and aortic valve pathologies^[11]^. However, the specific impact of LVEDD has not been thoroughly examined. Therefore, we conducted a pilot study to test the hypothesis that there exists a relationship between LVEDD and aortic expansion after AVR within the first year following the procedure.

## METHODS

In a large tertiary cardiology center, a longitudinal, prospective, non-concurrent, non-randomized unicentric trial was conducted on patients who underwent AVR between January 2021 and December 2022. The study included patients who received either a mechanical or biological prosthetic aortic valve. Data collection primarily relied on reviewing electronic medical records, supplemented by physical records when necessary. No direct contact with patients or interference in their treatment occurred, thus informed consent was waived. The study received approval from the hospital’s institutional review board (2022-VUSCH), and all participants in research-based studies provided informed consent.

All procedures followed were in accordance with the ethical standards of the responsible committee on human experimentation (institutional and national) and with the Helsinki Declaration of 1975, as revised in 2008.

During the specified period, a total of 125 patients underwent AVR at our institution. Among them, 62 patients were excluded as they did not meet the inclusion criteria, resulting in a final analysis of 63 patients. The selection for AVR was based on the patient’s symptoms and LV changes, following the guidelines of the European Society of Cardiology/European Association of Cardio-Thoracic Surgery^[2]^. All AVR procedures were performed via median sternotomy using cardiopulmonary bypass with moderate hypothermia. Additionally, 26 patients underwent combined coronary artery bypass grafting. Patients with well-controlled hypertension maintained stable blood pressure throughout the study. Exclusion criteria encompassed patients with pacemakers, cardiac resynchronization, or implantable defibrillators, hypertrophic cardiomyopathy with or without outflow tract obstruction, myocardial infiltrative disease, predominant AR, infectious endocarditis, prior aortic prosthesis (mechanical or biological), significant LV dysfunction (EF < 20%), perioperative deaths, and those lacking preor post-valve replacement echocardiogram data.

### Routine Echo Analysis

**Fig. 1:**
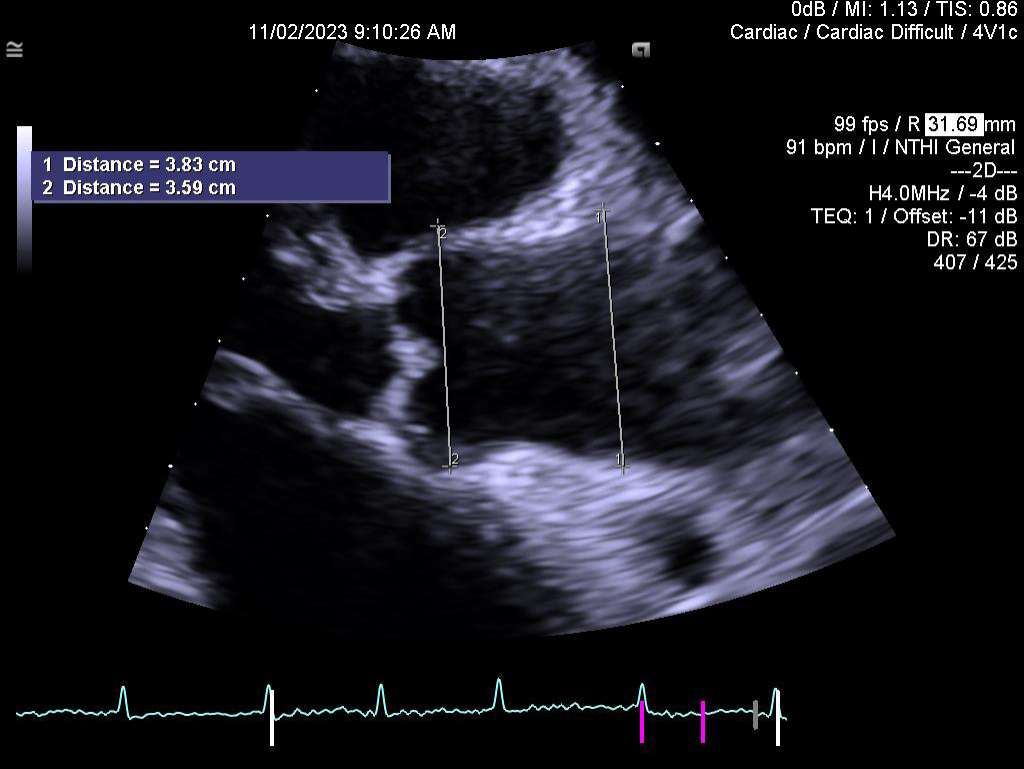
Measurement of the ascending aorta at the level of aortic root and sinotubular junction.

**Fig. 2:**
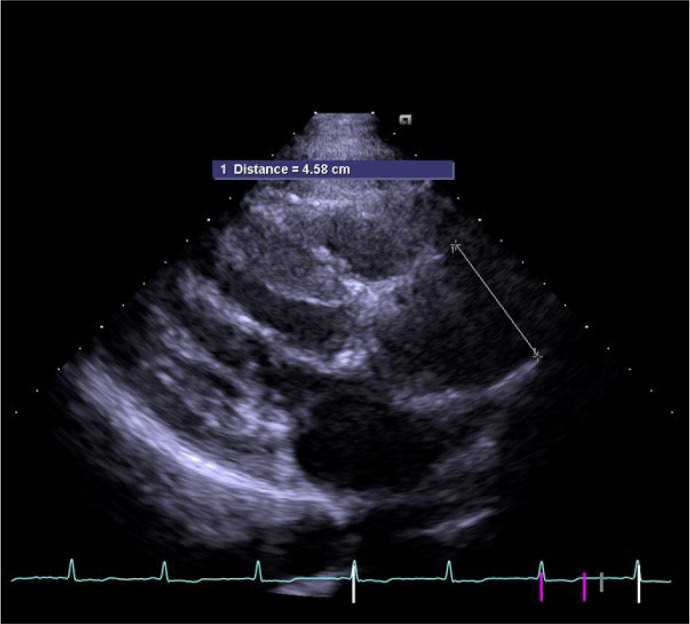
Measurement of the maximal dimension of ascending aorta.

All patients included in the study underwent a standard rest transthoracic echocardiography using Siemens ACUSON SC2000 Prime echo machines. Preoperative measurements of the ascending aorta, aortic root, EF, and LVEDD were obtained, as well as measurements one year after the operation. LV dimensions were assessed using bidimensional echocardiographic images in the parasternal long-axis view and M-mode. Echocardiographic LV volumes and EF were calculated using Simpson’s method with two apical views. Measurements of the ascending aorta were taken at three levels: aortic root, sinotubular junction, and the maximal dimension of the ascending aorta (Figures 1 and 2). Aortic sizes were determined in diastole using an inner wall to inner wall convention in the bidimensional parasternal long-axis view, with repeated cycles performed as necessary for accuracy. Mean and peak aortic gradients and flow velocity profiles were assessed using continuous wave Doppler measurements, and the native aortic valve orifice area was calculated using the continuity equation. The same measurement protocol was followed during the entire follow-up period.

### Statistical Analysis

Categorical variables were expressed as counts and percentages, while continuous variables were presented as mean ± standard deviation. Statistical analysis was performed using one-way analysis of variance. Student’s *t*-test was utilized to calculate *P*-values, and significance was defined as *P*<0.05. Correlations were determined using Pearson’s *r*. All statistical analyses were conducted using Prism 9.3.0 software (GraphPad Software, San Diego, California, United States of America).

## RESULTS

Table 1 displays the patients’ characteristics. The results indicate that there was a decrease in the dimensions of the ascending aorta and aortic root after AVR, although these differences were not statistically significant. There were no significant differences in LVEDD and EF before and one year after the operation (*P*=0.53 and *P*=0.65, respectively). Correlations were observed between the preoperative ascending aortic size and both the preoperative and one-year postoperative LVEDD (*r*=0.419, *P*=0.001 and *r*=0.320, *P*=0.314, respectively). After the operation, the correlation between the ascending aortic size and the preoperative and one-year postoperative LVEDD was slightly weaker (*r*=0.320, *P*=0.003 and *r*=0.136, *P*=0.335, respectively) (Table 2). There was no correlation found between the preoperative and one-year postoperative ascending aortic dimensions and the preoperative and one-year postoperative EF. Regarding the aortic root, a correlation was observed between the preoperative aortic root dimensions and both the preoperative and one-year postoperative LVEDD (*r*=0.452, *P*=0.001 and *r*=0.393, *P*=0.01, respectively) (Table 3).

**Table 1:** Patients’ clinical and echocardiographic characteristics.

Characteristic	
Age (n)	43.2 ± 13.34
Male sex (%)	32 (51)
Arterial hypertension (%)	26 (39)
Aortic regurgitation (%)	28 (44)
Aortic stenosis (%)	35 (56)
BAV (%)	8 (13)
TAV (%)	55 (87)
Combined CABG (%)	26 (41)
Mean gradient (mmHg)	53.65 ± 19.07
LVEF (%)	52.04 ± 8.85
LVEDD (mm)	51.56 ± 10.38
Ascending aorta (mm)	38.86 ± 11.53
Aortic root (mm)	35.77 ± 7.27
Biological valve (%)	17 (39.5)
Mechanical valve (%)	26 (60.5)
Prosthesis size 17-19 mm	3 (7)
Prosthesis size 21-23 mm	27 (63)
Prosthesis size 24-27 mm	10 (23)
Prosthesis size > 27 mm	3 (7)

BAV=bicuspid aortic valve; CABG=coronary artery bypass grafting; LVEDD=left ventricular end-diastolic diameter; LVEF=left ventricular ejection fraction; TAV=tricuspid aortic valve

**Table 2:** Statistical analysis of the preoperative and postoperative variables.

Variable	Preoperative value	1^st^ year	*P*-value
Ascending aorta (mm)	39.5 ± 8.20	37.82 ± 6.85	0.39
Aortic root (mm)	36.13 ± 7.46	34.48 ± 6.49	0.42
LVEDD (mm)	51.8 ± 10.35	52.90 ± 5.17	0.53
LVESD (mm)	37.1 ± 10.82	39.0 ± 6.32	0.56
IVS (mm)	14.19 ± 2.45	14.9 ± 2.38	0.20
LVEF (%)	51.8 ± 8.86	51.11 ± 5.0	0.65

IVS=interventricular septum; LVEDD=left ventricular end-diastolic diameter; LVEF=left ventricular ejection fraction; LVESD=left ventricular end-systolic diameter

**Table 3:** Correlations between the variables.

	AoAa	AoBa	LVEDDa	EFa	AoAb	AoBb	LVEDDb	EFb
AoAa		0.71	0.41		0.57	0.59	0.32	
AoBa	0.71		0.45	0.27	0.58	0.66	0.39	
LVEDDa	0.42	0.45		0.06	0.32	0.45	0.38	
LVEFa							0.292	
AoAb	0.57	0.57	0.32			0.43		
AoBb	0.59	0.66	0.42		0.43		0.28	
LVEDDb	0.32	0.39	0.38			0.28		0.32
LVEFb			0.29	0.43			0.32	

There are only significant correlations (< 0.001, < 0.01, < 0.05) in the table, data are shown as correlation coefficients *r*

AoAa=ascending aortic dimension before operation; AoAb=ascending aortic dimension one year after operation; AoBa=aortic bulb dimension before operation; AoBb=aortic bulb dimension one year after operation; LVEDDa=left ventricular end-diastolic diameter before operation; LVEDDb=left ventricular end-diastolic diameter one year after operation; LVEFa=left ventricular ejection fraction before operation; LVEFb=left ventricular ejection fraction one year after operation

## DISCUSSION

The objective of this pilot study was to investigate the relationship between preoperative and postoperative aortic size (ascending aorta, aortic root) and LVEDD in patients undergoing AVR. Our results indicate a strong correlation between these echocardiographic parameters, which serve as reliable indicators of successful AVR. Therefore, it is recommended to perform AVR before LVEDD increases, as this may be associated with expansion of the ascending aorta and aortic root, especially in patients with preexisting aortic dilation. Early AVR also reduces the risk of postoperative complications and mortality by preventing LV dilatation. Regular follow-up measurements are crucial to monitor any increase in LVEDD, which could indicate enlargement of the ascending aorta and aortic root after AVR.

Our study did not find significant differences in LVEDD and EF before and one year after AVR, which is consistent with a study by Joaquim et al.^[12]^ where the second echocardiogram was conducted one year after AVR. They also observed a decrease in LVEDD and an increase in EF in the first echocardiogram performed within the first six months after surgery, which was statistically significant. Between the two measurements, LVEDD increased, and EF decreased. In our study, measurements were only taken one year after surgery. Another study by Naicker et al.^[13]^ demonstrated a significant postoperative increase in LVEF and a non-significant decrease in LVEDD during follow-up echocardiograms performed at an average of 610 ± 123 days after surgery. These findings are consistent with a meta-analysis by Perry and Li, where LVEF was associated with the effect of AVR and vice versa^[14]^.

Regarding the aorta, we observed a significant decrease in the diameters of the ascending aorta and aortic root one year after AVR. Our patient cohort consisted of individuals with and without aortic dilation, with the majority having tricuspid valve morphology and only a few with a bicuspid aortic valve. A study by Nitsche et al.^[15]^ demonstrated that in patients with a baseline aortic dilation > 4 cm, the aortic diameter decreased during follow-up, and larger baseline aortic diameters were associated with smaller postoperative annual aortic expansion rates. Similarly, Zhang et al.^[6]^ found that in the AVR alone group, the median aortic expansion rate was -0.66 mm/year, and the aortic expansion rates were not influenced by aortic valve morphology (bicuspid *vs.* tricuspid) or initial aortic diameter. They compared different management strategies for dilated ascending aorta. Furthermore, Banovic et al.^[16]^ reported that patients with bicuspid or tricuspid aortic valve stenosis and mild to moderate ascending aortic dilation (40-50 mm) had a comparably low risk of adverse aortic events (aortic diameter expansion, aortic dissection) 15 years after isolated AVR.

### Limitations

Our study has several limitations that should be considered when interpreting the results. Firstly, the study was conducted at a single center, which may limit the generalizability of the findings. Additionally, the retrospective nature of the study design introduces potential biases, such as patient selection bias. The reliance on available echocardiograms, which were sometimes incomplete for a comprehensive assessment of ascending aorta and LV dimensions, is another limitation. However, this can also be seen as a strength, as it reflects real-life standard echocardiograms. Furthermore, the inclusion of patients with both aortic dilatation and without aortic dilatation may have influenced the results, as the effect of aortic wall pathology on the outcomes could not be specifically studied. Similarly, the inclusion of patients with both bicuspid and tricuspid aortic valves suffering from AS and AR did not allow for an independent analysis of the specific effects of aortic valve morphology and pathology. Lastly, the small sample size of the study limits the statistical power and increases the risk of Type II errors. To establish the generalizability of our findings, further longitudinal studies with larger sample sizes are needed to confirm and expand upon our results.

## CONCLUSION

While our study indicates a notable association between preoperative and postoperative aortic size (aortic root, ascending aorta) and postoperative LVEDD, it is important to note that these findings should be interpreted with caution at this early stage. Additional research is needed to validate and fully understand the implications of these relationships. Furthermore, longitudinal studies are warranted to assess the clinical significance and potential applications of our findings in the management of patients undergoing AVR.
